# P-706. Adenovirus Type 21 Outbreak among Haematology and liver transplant patients at a large tertiary care hospital in Singapore

**DOI:** 10.1093/ofid/ofae631.902

**Published:** 2025-01-29

**Authors:** Mabel Z Q Foo, Darius Y W Chan, Indumathi Venkatachalam, Hei Man Wong, Jean Xiang Ying Sim, Deborah C M Lai, Lin Cui, Moi Lin Ling

**Affiliations:** Singapore General Hospital, Singapore, Not Applicable, Singapore; Singapore General Hospital, Singapore, Not Applicable, Singapore; Singapore General Hospital, Singapore, Not Applicable, Singapore; Singapore General Hospital, Singapore, Not Applicable, Singapore; Singapore General Hospital, Singapore, Not Applicable, Singapore; Singapore General Hospital, Singapore, Not Applicable, Singapore; NPHL, NCID, Singapore, Not Applicable, Singapore; Singapore General Hospital, Singapore, Not Applicable, Singapore

## Abstract

**Background:**

Human adenoviruses (HAdVs) commonly cause acute respiratory illness, with documented outbreaks in military settings. HAdV infections frequently precipitate severe pneumonia amongst immunocompromised patients, with associated mortality risk. We report a HAdV type 21 infection cluster involving immunocompromised patients at a tertiary care hospital.

**Figure:**
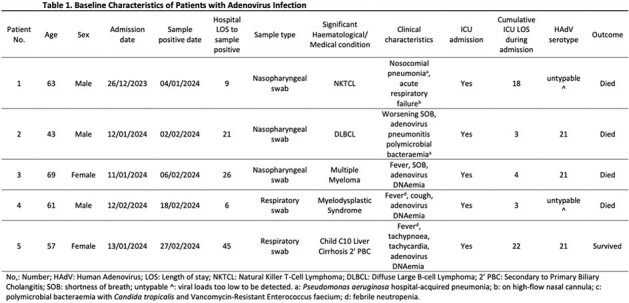

**Methods:**

Hospital-onset (HO) adenoviral infection was defined as positive HAdV polymerase chain reaction (PCR) test with corresponding respiratory symptom onset six days after admission. Positive samples were typed in the National Public Health Laboratory by sequencing hexon gene variable regions. Epidemiological investigations were carried out for potential spatiotemporal linkage between these patients for targeted infection prevention interventions.

**Figure:**
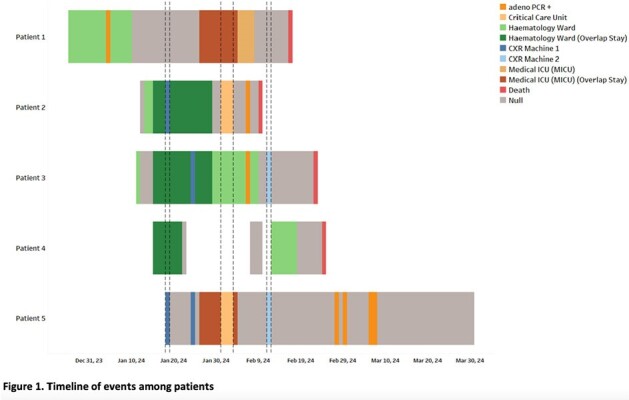

**Results:**

Between 1^st^ January to 31^st^ March 2024, 21 HAdV-positive cases were identified, with five categorised as HO HAdV. Four patients (80%) had haematological malignancies and one had received a liver transplant 23 days prior to testing positive for HAdV. The mean age of patients was 58.6 years (SD: ± 9.7), with males comprising 60% of the cohort. The median hospital length of stay (LOS) before HAdV positivity was 21 days (range: 6 – 45). Clinical manifestations included fever, shortness of breath, cough, and acute respiratory failure. All patients required Intensive Care Unit (ICU) admission, with an average ICU LOS of 10 days (SD: ± 9.2). Respiratory samples of three patients were typed as HAdV 21, and three had adenovirus DNAemia.

In the identified cluster, the four patients with haematological malignancies shared the same ward, with two overlapping in time but housed in different rooms. The liver transplant patient had overlapping stay in the critical care unit with a patient from the haematology cluster and shared the same mobile chest x-ray machine with two others from the same cluster. All four patients with haematological malignancies experienced same-admission mortality.

**Figure:**
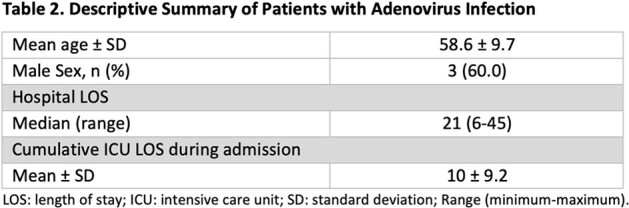

**Conclusion:**

Timely symptom recognition and respiratory multiplex panel testing enabled detection of HAdV infection cluster in immunocompromised patients. Early cluster detection and reinforcement of infection prevention measures at risk locations can mitigate transmission of HAdV infections.

**Disclosures:**

**Moi Lin Ling, FRCPA**, Solventum: Honoraria|Solventum: Educational grant for APSIC projects

